# INFLUENCES ON CAREGIVERS OF OLDER VETERANS’ PERCEPTIONS OF THEIR QUALITY OF LIFE: A NATIONAL SURVEY

**DOI:** 10.1093/geroni/igae098.1236

**Published:** 2024-12-31

**Authors:** Lauren Moo, Elizabeth Chamberlin, Victoria Ngo, Steven Shirk

**Affiliations:** Department of Veterans Affairs, Bedford, Massachusetts, United States; Department of Veterans Affairs, Bedford, Massachusetts, United States; Department of Veterans Affairs, Bedford, Massachusetts, United States; Department of Veterans Affairs, Bedford, Massachusetts, United States

## Abstract

Being a family caregiver can negatively impact an individual’s physical and mental health, leading to caregiver burden, burnout, and lower quality of life (QoL). We explored caregiver and care recipient factors that influence caregiver QoL via a national web-based survey of 511 informal caregivers of older Veterans. We found that greater dependence of the Veteran on the caregiver for Activities of Daily Living (ADLs) and Instrumental Activities of Daily Living (iADLs) was associated with poorer QoL (p<0.001). Caregivers’ cohabitation with the Veteran was also associated with poorer QoL (p<0.001), while caring for an additional person was not. However, our results also suggest that caregivers making time for their own medical care and social needs were unique important predictors of higher caregiver QoL, and this positive benefit remains even when considering the negative impact of Veteran ADL and iADL support needs. In fact, our results suggest that making time for social activities is the strongest predictor of QoL. Thus, making time to receive medical attention and participating in social activities are important factors when considering the caregivers’ overall QoL. Cohabitating with the care recipient may be required for various reasons (e.g., amount of care needed, financial limitations) and, therefore, may be challenging to address. However, improving and supporting the amount of self-care the caregiver receives may be a feasible area to address and can still have a substantive impact on their overall QoL.

